# Genetically-encoded sensors of protein hydrodynamics

**DOI:** 10.18632/oncotarget.4785

**Published:** 2015-07-03

**Authors:** Alexander Hoepker, Yuling Yan, Gerard Marriott

**Affiliations:** University of California, Berkeley, Berkeley, CA, USA

A major challenge in cell biology is to understand the molecular basis of complex processes, including motility and cytokinesis. Meeting this challenge will require new genetically-encoded fluorescent sensors that allow for quantitative, sensitive, dynamic and high-resolution imaging of signaling proteins and their effector protein complexes in living cells. Currently, the most popular sensors are designed to undergo a change in Förster resonance energy transfer (FRET) on binding to their molecular target. A more direct approach to quantify target proteins in a cell would employ a fluorescence anisotropy (FA) sensor that is sensitive to the increase in molecular volume or hydrodynamics in the target-bound complex. Until our recent work (Hoepker et al, 2015) [1], the special physical and photo-physical properties required for sensors of protein hydrodynamics were only known for synthetic fluorophores.

In spite of their popularity, genetically-encoded FRET-sensors based on CFP and Venus suffer from the large size of each protein, which reduces the difference in FRET efficiency between the free and target-bound states. The distance between the two dipoles for the CFP-Venus FRET pair at 50% FRET efficiency (R_0_) is 5.4 nm, and the maximum FRET efficiency for CFP directly connected to Venus in a fusion protein is 31% (1.14 R_0_) [2], which corresponds to a distance of ~6.2 nm. CFP and Venus are actually closer together than 6.2 nm, as this distance includes an offset of at least 2.5 nm that comes about because the center of the buried dipole in CFP and Venus is at least 1.25 nm from the protein surface. The incorporation of the capture sequence (25 kD~50 kD; φ of 3~4 nm) between CFP and Venus will increase the distance between the dipoles to 1.5R_o_~2R_o_ with transfer efficiencies hydrodynamics and molecular volume. The specificity of 8%~1.25%, respectively. Accurate measurement of FRET efficiencies of this magnitude based on the change in the fluorescence signal of CFP is difficult to perform in cells, and so the FRET signal is usually presented as the ratio of the sensitized acceptor and donor signals. The ratiometric measure is sensitive however to non-FRET events that include photo-bleaching of endogenous molecules whose emissions overlap with the donor probes, and by excitation-induced maturation of acceptor probes that may increase the acceptor signal.

Carefully designed FA-sensors can overcome many of the limitations of FRET sensors. FA-sensors are in principle easier to design, as they are composed of a single fluorescent probe that is linked to a specific capture sequence. In addition the FA value associated with target-binding scales predictably with the molecular volume of the sensor-target complex. Finally, the FA values of the free and target-bound states of a FA-sensor may differ by a factor of two with intermediate values being proportional to amount of bound target. In spite of these highlighted advantages, FA is rarely used to quantify target proteins; one reason being that CFP and GFP have FA values close to the theoretical limit, a consequence of their large mass (28 kD) and short fluorescence lifetime (2.25 ns). The breakthrough in the design and optimization of genetically-encoded FA-sensors of protein hydrodynamics (Hoepker et al, 2015) came about by exploiting the long lifetime (13.6 ns) and small mass (20 kD) of the lumazine-binding protein (LUMP). The FA value of LUMP (0.167) is similar to that calculated for a 20 kD sphere, and less than half of the theoretical maximum value of 0.35. The FA value of a LUMP fusion with the 5 kD G-protein binding domain (GBD) is 0.176, and increases to 0.207 in the complex with GTP-Rac1, consistent with the FA-value calculated for a 41 kD sphere. The error in the FA measurement is +/−0.001, which allows us to append capture sequences of ~70 kD to LUMP and still generate significant statistically significant differences in FA for the target-bound sensor. Finally, images of the FA-value of LUMP can be recorded using a fluorescence microscope equipped with polarizers– the resultant high-spatial resolution images of FA can serve to resolve the distributions of free LUMP-GBD and its complex with a constitutively active Rac1 in living cells.

LUMP is unique amongst genetically-encoded fluorescent proteins in being sensitive changes in protein of the common LUMP sensor for any molecular target is achieved by appending an appropriate capture sequence to the N- or C-terminus. This simple design can be extended to generate a library of LUMP-based FA-sensors with each member harboring a sequence that is unique to each protein in an organism. The development of new genetically-encoded FA-sensors [1] provides an attractive alternative to FRET-based approaches for high-throughput screening, and for quantitative imaging and analysis of target molecules in biological systems.
